# African American Cancer Caregivers’ Decision on Palliative Care Use: A Social Ecological Perspective

**DOI:** 10.1093/geroni/igaf122.3527

**Published:** 2025-12-31

**Authors:** Moses Akwobugi, Elisha Oduro

**Affiliations:** University of Maryland Baltimore, Baltimore, Maryland, United States; University of Maryland Baltimore, BALTIMORE, Maryland, United States

## Abstract

African American (AA) cancer caregivers are less likely to pursue palliative care for their relatives compared to Whites leading to differences in cancer outcomes. However, little is known about the multi-level influences on AA cancer caregivers’ palliative care decision-making. Using the Social Ecological Model as a conceptual lens, this study develops a theoretical framework to understand the multi-level influences on African American cancer caregivers’ palliative care decision-making. The model highlights five levels of palliative care decision-making influences: intrapersonal, interpersonal, organizational, community, and policy. At the intrapersonal level, health literacy and education level influence how AA cancer caregivers perceive the purpose and benefits of palliative care. Interpersonally, family dynamics and trust in clinicians influence their willingness to engage in shared decision-making about palliative care. Organizationally, the availability of culturally sensitive palliative care services and clinicians’ culturally aligned communication influences their palliative care acceptance. Community partnerships with churches and faith leaders validate palliative care as consistent with cultural and spiritual traditions, influencing their palliative care acceptance. At the policy level, strengthening Medicare coverage for palliative care services and affordable community-palliative care programs reduces cost barriers and improves access, influencing AA cancer caregivers’ palliative care decisions. This framework underscores the need for coordinated, multi-level palliative care interventions to support AA cancer caregivers to make informed palliative care decisions and reduce disparities in cancer outcomes. Further, it provides a theoretical foundation for future research and practice aimed at improving palliative care use in AA communities.

